# What Is Artificial about Life?

**DOI:** 10.1100/tsw.2011.73

**Published:** 2011-03-07

**Authors:** Alessandro Giuliani, Ignazio Licata, Carlo M. Modonesi, Paolo Crosignani

**Affiliations:** ^1^ Environment and Health Department, Istituto Superiore di Sanità, Roma, Italy; ^2^ Institute for Scientific Methodology, Palermo, Italy; ^3^ Museum of Natural History, Evolutionary and Functional Biology Department, University of Parma, Italy; ^4^ Cancer Registry and Environmental Epidemiology Unit, National Cancer Institute (INT), Milano, Italy

**Keywords:** reductionism, vitalism, synthetic biology, technoscience, theoretical biology

## Abstract

The announcement of “Artificial Life” by the Craig Venter group, and the media stir that arose from the news, provoked thoughts about the current technologies in contemporary science and the cultural tension of such projections on the media. The increasingly blurred boundaries between specialist and generalist media, while promising a wider appreciation of scientific discovery, potentially allow unrealistic, ideological claims to dictate scientific research. This is particularly evident in biology, where the pervading paradigm is still dominated by a physically naïve reductionism in which the only relevant causative layer is the molecular one. The reductionist hypothesis is that everything one observes is the result of an underlying molecular mechanism almost independent of the context in which it operates. Molecular mechanisms are often necessarily studied in isolation and therefore operate in unnatural conditions. The mechanistic view of biological regulation implies that we think of genes as intelligent agents. Here we try to critically analyze the motivations behind the spread of such unrealistic simplifications.

In reading the Venter group's original paper on 'Creation of a Bacterial Cell Controlled by a Chemically Synthesized Genome'[1]**,** we find out that a sequence of DNA engineered *a priori* by "copying" it from a natural organism, a *Mycoplasma*, was inserted into a bacterium. This means that the genetic information in this extremely simple genome was not written *ex novo.*

It is undoubtedly a remarkable technological advancement, but we can speak neither of "artificial life", nor of a revolutionary method of chemical synthesis at all. So, let us try to clarify the whole matter further so as to understand what belongs to invention, what to media hype, and how such claims impact current ideas of biological systems. We think this exercise has merit, especially now that the media "bubble" of the Venter group's discovery has faded away and we are in a good position to comment objectively.

The "copying and reproducing" mechanism driven by the DNA base-pairing rules is crucial for life, allowing for both the reproduction of identical cells and the production of RNA molecules (that, in turn, are the basis for protein synthesis) with very high fidelity. Nevertheless, a molecule is not a living being. A molecule does not produce and does not do anything by itself; it does not use the energy of the environment to build its constituents, it does not develop, and it does not die (it degrades, instead). On closer inspection, it does not duplicate either, because the processes described above require the combined working of sophisticated chemical machines (enzymes, structural proteins, supramolecular complexes) in order to be completed.

Conversely, a bacterium is a living being. It has a membrane with a very complex structure that separates it from the external environment, a distinctive trait that makes a bacterium a self-regulating biological system that struggles for survival, reproduces, and finally dies. For all these reasons, some molecules will freely go in and out of the system, others will be accumulated, and still others will be produced or destroyed. This takes place thanks to a complex molecular architecture that allows for an efficient metabolism, a process otherwise physically impossible. Claiming that one has created artificial life only because a “stylized” genome has been inserted into a bacterium deprived of its own genome sounds like a somewhat exaggerated claim if not actually absurd.

We have to be clear on this point: a “centralist” conception where DNA is regarded as the sole causal principle of life is beyond any scientific evidence. It is not a matter of arguing between scholars whose viewpoints are different, it is a matter of basic biology on which all scientists completely agree. There is no need to introduce the central idea of systems biology. Denis Noble has clearly pointed out[2]: *“Where is, if any, the program of life? My thesis is that there is no program and that, in biological systems, there is no privileged level of causality”.* In conclusion, looking for a conductor is useless; it is the orchestra itself playing here!

How and why do such naïve simplifications spread? According to other authors (e.g., Richard Lewontin[3]), many scientists talk about DNA as the “program of life” because they believe that all biological attributes of an organism are prefigured in its genes and all that is required for a fixed output is for the *enter* key to be pressed. However, this point of view is not exhaustive of the subject matter and we need some other explanations. The first explanation could be economically grounded: after the wondrous promises of the 1980s, biotechnology slumped. Thus, researchers lured investors with the idea of complete control on the living. Adam Wilkins’ paper is illuminating[4]: *“Maybe the sheer novelty of the news is just considering a novelty the fact that genes work within complex regulatory networks. How so many people involved in biotechnology could have neglected an idea (combined action of genes,* Editor’s note) *which is a proved fact in developmental biology and many other fields for decades?”*

And yet the centralist idea of genome still supports questionable (at least) projects.

In *Nature*[5], you can find a paper on a mega-project – hundreds of millions of dollars per year – to find *“each gene’s role”* by knocking out one gene at a time from a group of mice and then checking for differences compared with the wild type. Not only do we forget what Wilkins has said – genes work together – but we forget common sense too: if I remove a spark plug, my car will not run properly, but it will never cross my mind to consider a spark plug as the car engine (or try to understand how a car runs from the structure of a spark plug).

Three main principles, in our opinion, are at the basis of this kind of project:

- The myth of a big enterprise that mobilizes a great deal of resources regardless of the initial aim and thus creates an important spill-over effect simply because it put together “thousands of brains”. It is the classical theme of the Manhattan Project, space enterprise, big accelerators, the mathematical theology of the Theory of Everything, and – of course – DNA decoding. These are the “magnificent progressive expectations” of science in search of consent and funding, based on the misleading idea of innovation as the naïve never-ending development of technology by means of Lego®-like conceptual bricks that are made of already-given knowledge.
- The myth of ruling over Life by removing any risk, disease, and unpredictable event. During the last half of the century, basic research acquired more and more clues that provided evidence that most of what we observe in living beings has a multifactorial and, thus, a probabilistic nature. Applied research, instead, focused on DNA manipulation with the idea that any biological process is directly, linearly, and irreversibly gene specified. The recent achievements in molecular biology – *in primis* Human Genome Project (to which Venter greatly contributed)[6,7]

– have definitely shown that DNA is only one of the factors determining the phenotype of organisms. Genes are less numerous than proteins. Thus, it is patent that the molecular machine can modify or modulate gene expression according to how the cell decides to react to environmental stimuli; not to mention, different kinds of biological inheritance (e.g., epigenetic inheritance).

- Connected with the two points stressed above, there is a third point much more important for its implications in public health. There is growing evidence to suggest that biomedical practices to predict the genetic susceptibility to cancer and other diseases have very weak significance apart from a very few cases (e.g., the genetic risk of breast and ovarian cancer associated with variants in BRCA1 and BRCA2 genes). Even the prediction of an individual's probability of cancer has led to useless results both in terms of the probability itself (rarely greater than 10% of extra risk) and in terms of consistency of results[[8]. Any illusory mechanism aimed to control biological systems starting from genetic knowledge is simply unrealistic, for rather obvious physical considerations.

Notwithstanding, if we try to abandon the classic approach based on fully linear and deterministic sequences of molecular events, we should expect to hear ourselves labeled as "mystics" or — there is perhaps no more worrisome epithet within biology today — "vitalists"[9].

Curiously enough, it is exactly the opposite: describing cellular processes (e.g., Krebs cycle, lipid biosynthesis) as they were made, as an ordered series of single events, postulates an invariant series of singularly independent "encounters" between intervening molecules. This is impossible to understand without considering biological molecules as "intelligent agents", i.e., without acquiring a fully "vitalist" attitude that calls for a suspension of physical laws in the biological realm. In order to reconcile facts and physically plausible models, it is mandatory to make use of concepts such as form and "structured phases", imposing a general order to the biological systems in which the single molecular events are no more considered as separate diffusion-driven events[10,11], but are orchestrated into fully connected wholes. On the contrary, the word "mechanism" suggests the mechanical nature of the studied phenomena and, thus, the presence of linear causal links that, given their huge number, must be necessarily ordered by a "molecular intelligence". This molecular intelligence should be mandatory to explain the presence of very precise metabolic pathways involving very long sequences of specific events (e.g., chemical reactions), if we maintain the hypothesis of a fully random phase in which the cellular biochemistry takes place. The presence of a general order in the form of a sort of "solid-state" (formed, ordered phases) arrangement could reconcile the presence of organized molecular pathways with known physical principles.

Actually, such a modern "Laplace's Demon"[12] shows insurmountable conceptual limits, as in physics — as the beautiful analyses of mesoscopic phenomena by Anderson[13] and Laughlin and Pines[14] have pointed out — and it is completely unfit for life sciences. The problem with a "physically realistic" picture of biology in which nonlinear and network-like properties are seriously taken into account comes from the fact that biological systems can no more be considered as "completely controlled and modifiable", but they can only assume some "energetically allowable" functioning modes (attractors) that drastically limit our intervention possibilities[15].

We must be fully aware that beyond any technology, there is an epistemological choice in terms of underlying physical model, to be evaluated critically and carefully. Paradoxically, in the age of extreme individualism, there is no place for the complexity of the living.
